# Supplementary material for: Fast kinetics of magnesium monochloride cations in interlayer-expanded titanium disulfide for magnesium rechargeable batteries
Source: Nat Commun. 2017 Aug 24;8:339. doi: 10.1038/s41467-017-00431-9 (PMC5569106; doi:10.1038/s41467-017-00431-9)
Supplement: Supplementary file 2 — Supplementary Information [file 41467_2017_431_MOESM2_ESM.pdf]

Description of Supplementary Files

File Name: Peer Review File

File Name: Supplementary Information
Description: Supplementary Figures, Supplementary Tables, Supplementary References.

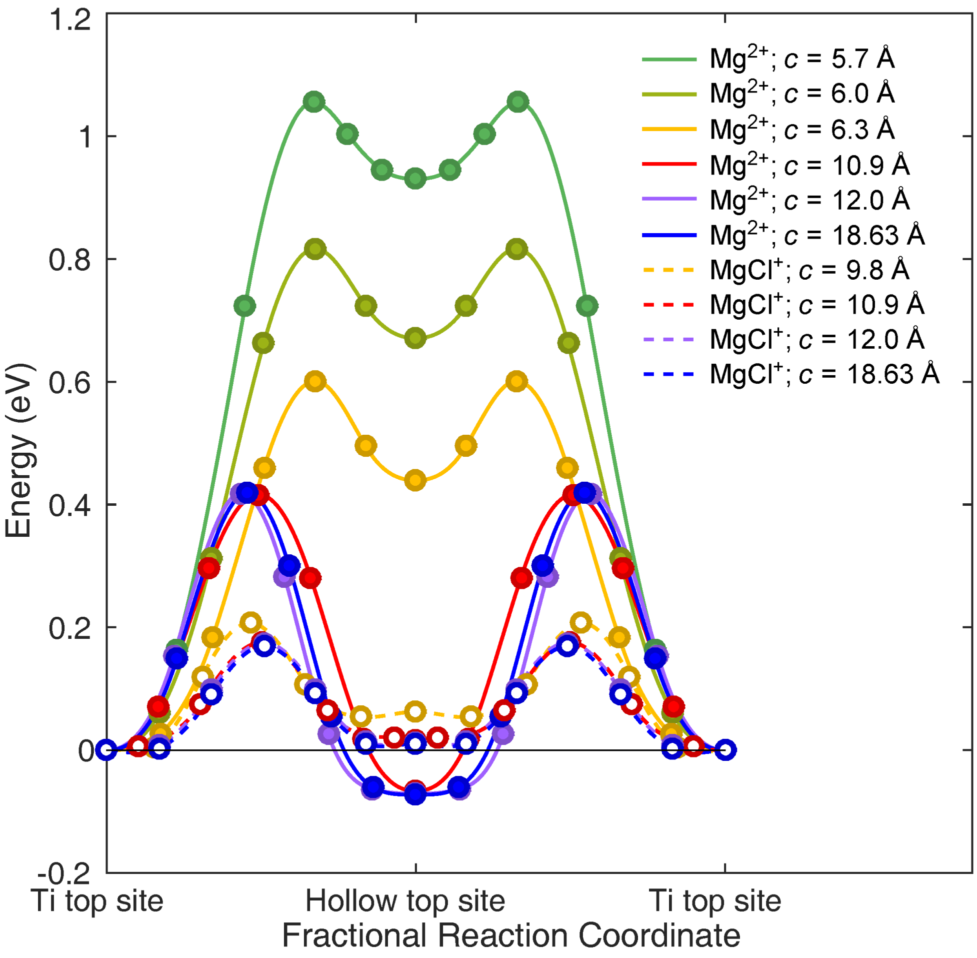

Supplementary Figure 1. Minimum energy pathway of Mg^{2+} and $MgCl^{+}$ in 1T-TiS_2 at the dilute limit with different *c* lattice parameters. The migration barrier converges to the lowest value at 10.9 Å, and the barrier could not be lowered anymore by further expanding the interlayer distance larger than 10.9 Å (e.g., to 18.63 Å). The energy barrier values of Mg^{2+} in pristine TiS_2 are in good agreement with those recently reported by Van der Ven *et al.*[1]

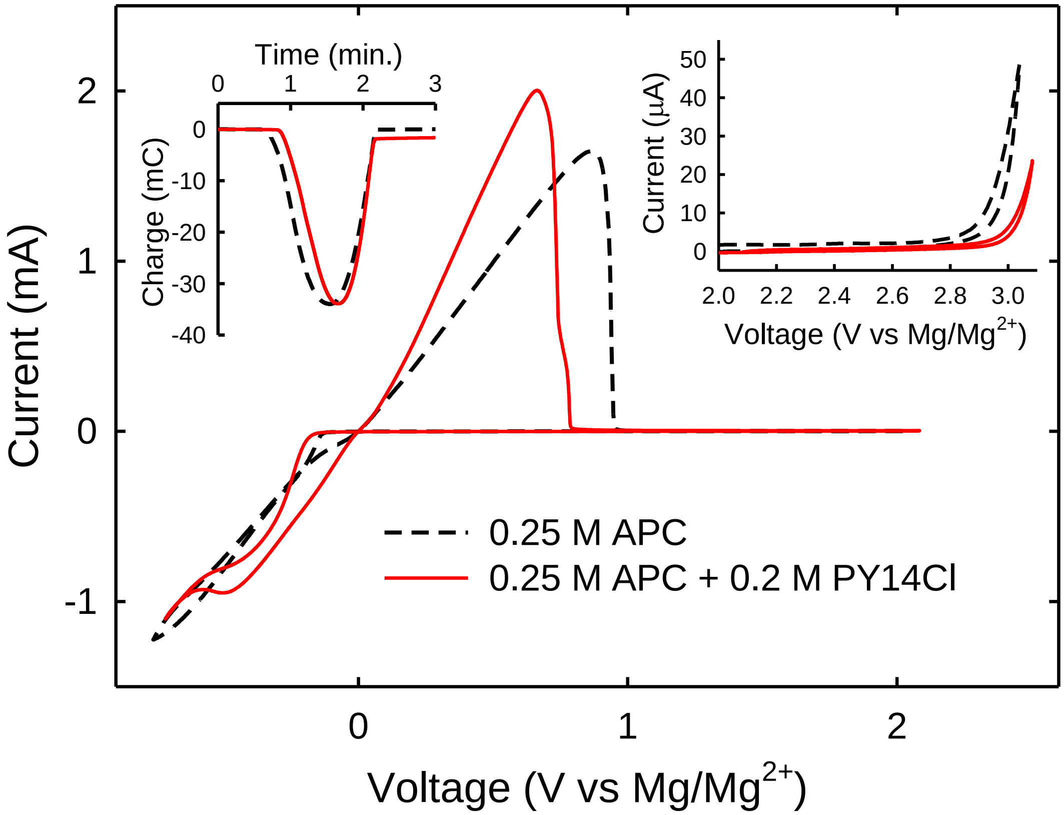

Supplementary Figure 2. Cyclic voltammograms of APC electrolytes with and without PY14Cl (25 mV s^{-1}). A platinum wire and two magnesium foils were used as the working, reference, and counter electrodes, respectively. A small increase in overpotential for Mg deposition from 0.14 to 0.16 V and a slight drop in coulombic efficiency from 100% to 95.2% were observed with the addition of PY14Cl to APC. Also, the overpotential of Mg dissolution is decreased from 0.86 to 0.67 V and the anodic voltage stability window is enhanced from 2.6 to 2.8 V vs Mg/Mg^{2+} by the addition of PY14Cl.

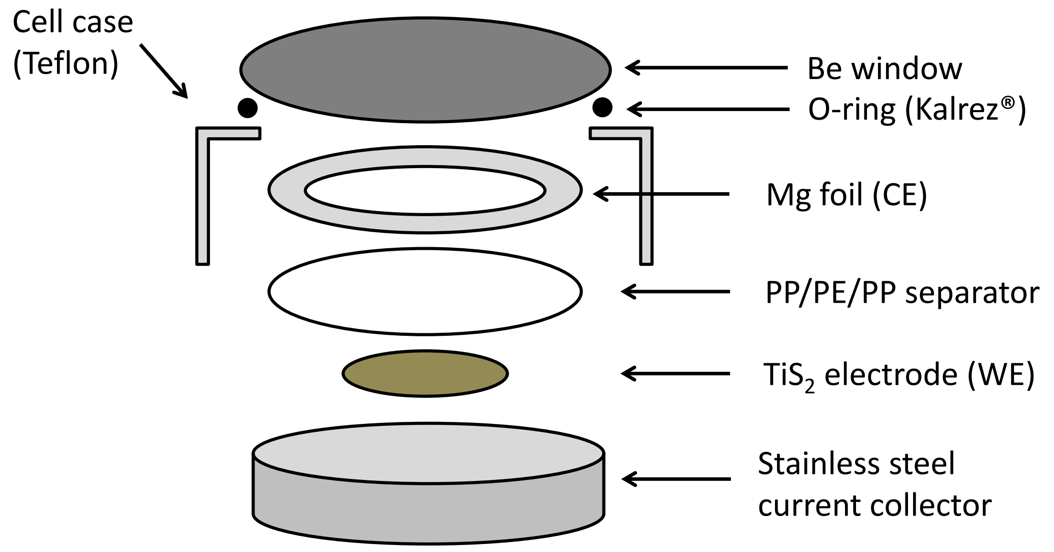

Supplementary Figure 3. Configuration of an *in operando* XRD cell. Mg metal anode was placed on the Be window to avoid electrochemical dissolution of Be metal. The center of Mg metal was punched out to enhance the intensity of X-ray that reaches to and returns from TiS_2. We placed Mg anode onto the Be window because Be metal undergoes electrochemical reaction at potentials higher than 0.53 V vs Mg/Mg^{2+} in APC electrolyte. In this configuration, Mg acts as sacrificial anode and Be is kept as Be^0 because of ca. 0.53 V lower standard electrode potential of Mg compared to Be. Similar configuration has been used by J. Dahn and co-workers to measure *in operando* XRD for a high voltage Li-ion cathode[2].

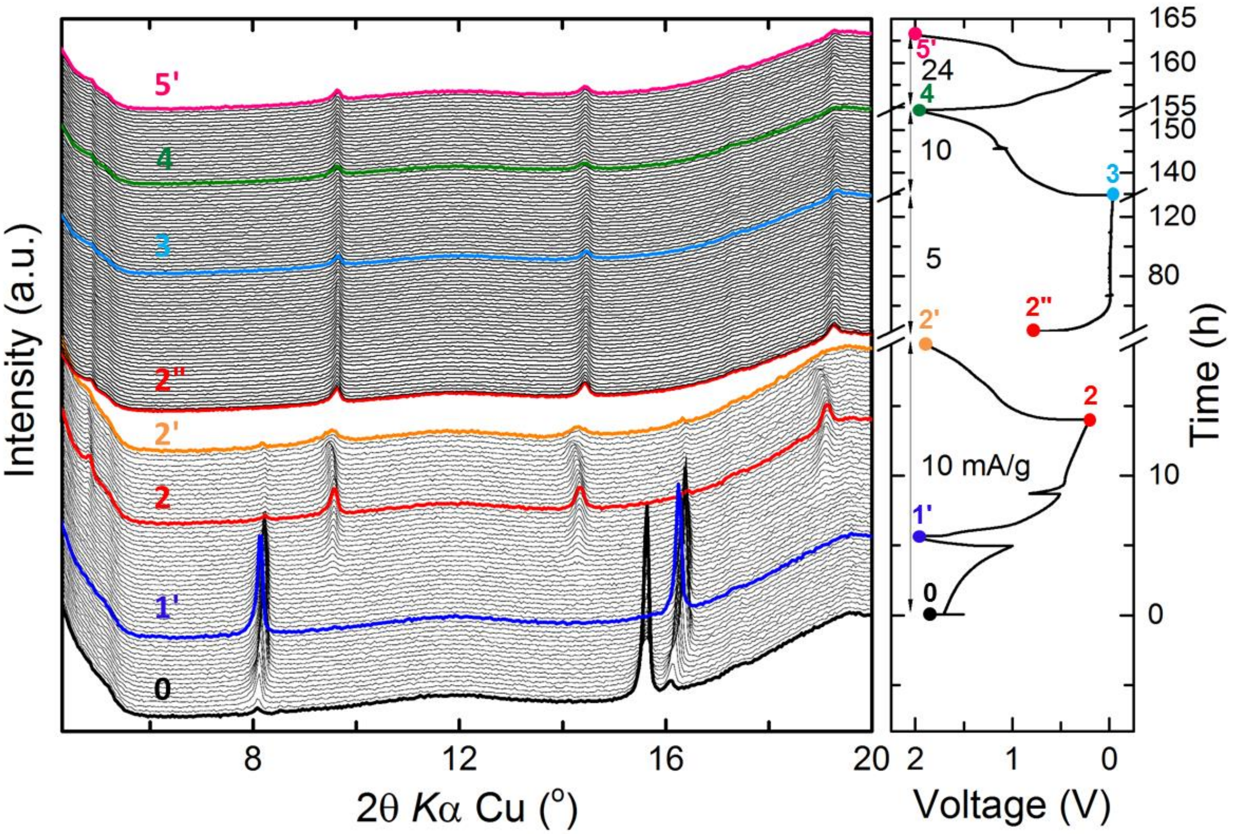

Supplementary Figure 4. Another set of *in operando* XRD patterns to check the structural irreversibility of each stage **1**, **2**, and **3**. The discharge cut-off voltage was controlled to 1.0, 0.2, and 0.0 V followed by charging back to 2.0 V to confirm the structural irreversibility of each stage **1**, **2**, and **3**. Prime (') denotes the charged state followed by a certain stage. Between stages **2**' and **2**'', the cell was cycled within 0.0 and 2.0 V at 10 and 24 mA g^{-1}, whereby the structural change was minimal. Note that the complete 4.74° peak was detected clearly in the diffraction patterns of stage **2** and the subsequent stages for this measurement.

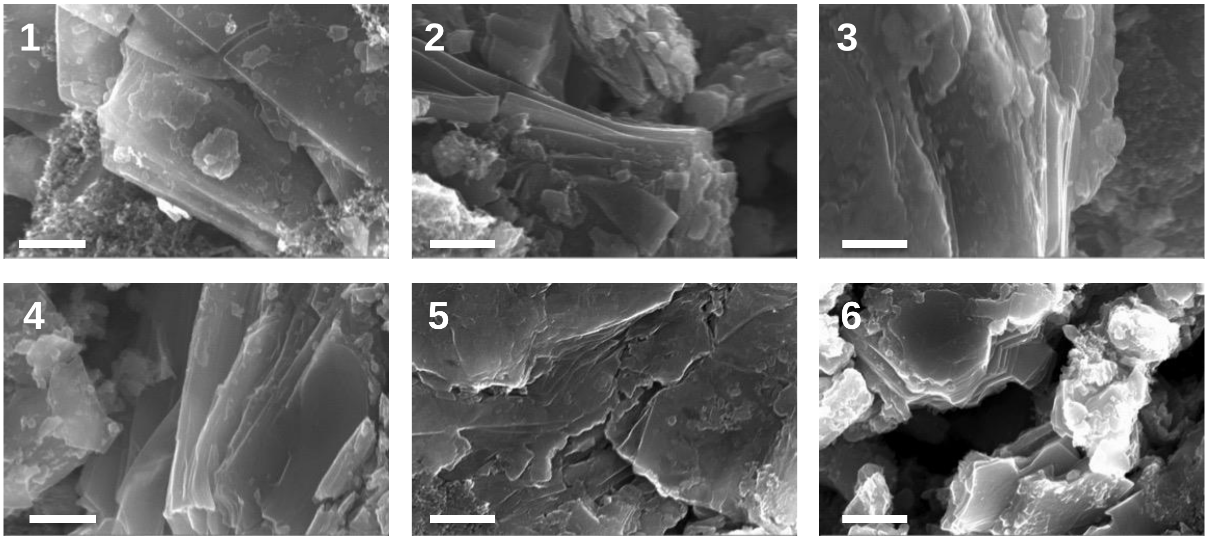

Supplementary Figure 5. FE-SEM images of TiS_2 electrodes at different stages **0-5** during the first discharge process. Scale bars: 2μm.

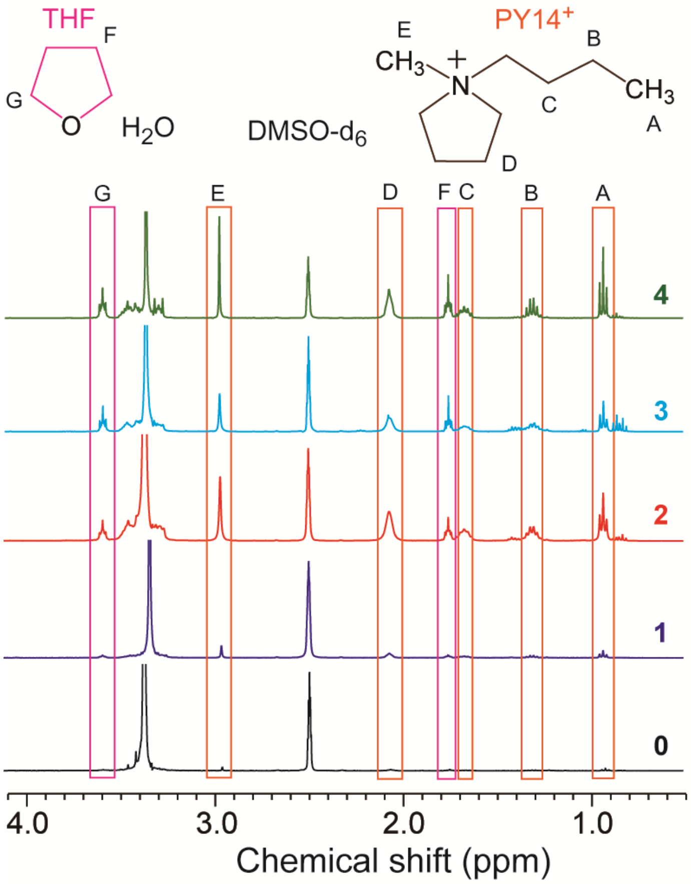

Supplementary Figure 6. NMR spectra of samples after sonication and heating in DMSO-d_6 solutions. Chemical shifts at boxes A, B, C, D, and E represent the proton of $PY14^+$ ion, while those at boxes F and G represent the proton of THF solvent. Chemical shift at 2.5 ppm and 3.4 ppm correspond to DMSO-d_6 and H_2O, respectively. Stage **0** shows negligible amount of $PY14^+$ and THF. Note that the sample at stage **0** was deliberately dipped in $PY14^+$ containing Mg-ion electrolyte and then rinsed with the same washing condition as the samples at stages **1**-**4**. That means the $PY14^+$ and THF signals at stages **1-4** do not come from the trace amount of salt or solvents that can be possibly remained after washing.

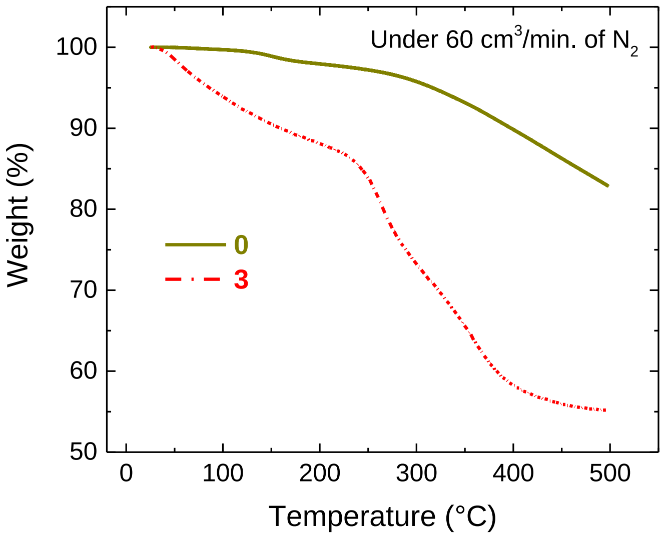

Supplementary Figure 7. TGA for the samples of stages **0** and **3** in nitrogen flow. Heating rate: 5 °C min^{-1}.

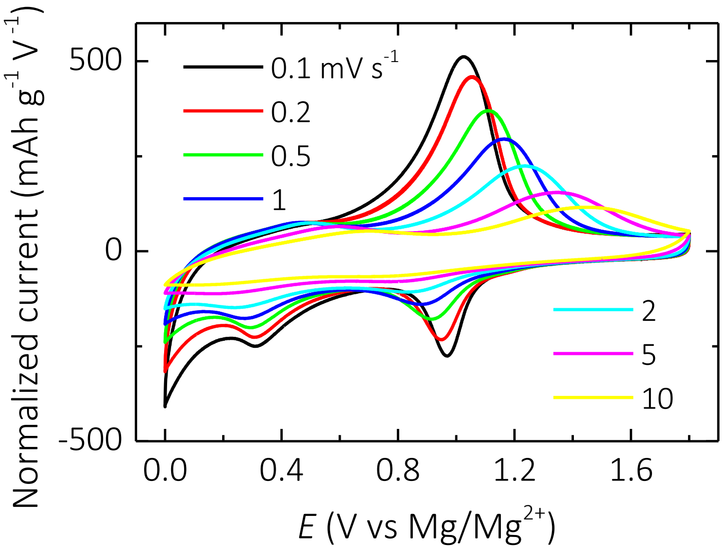

Supplementary Figure 8. Cyclic voltammogram of an expanded TiS_2 electrode at varied scan rates from 0.1 to 10 mV s^{-1}. The vertical axis shows current normalized by scan rate. Upon negative scan, at least two cathodic current peaks appear at 1.0 and 0.3 V vs Mg/Mg^{2+}. On the other hand, the corresponding anodic current peaks are merged at ca. 1.0 V vs Mg/Mg^{2+} upon positive scan.

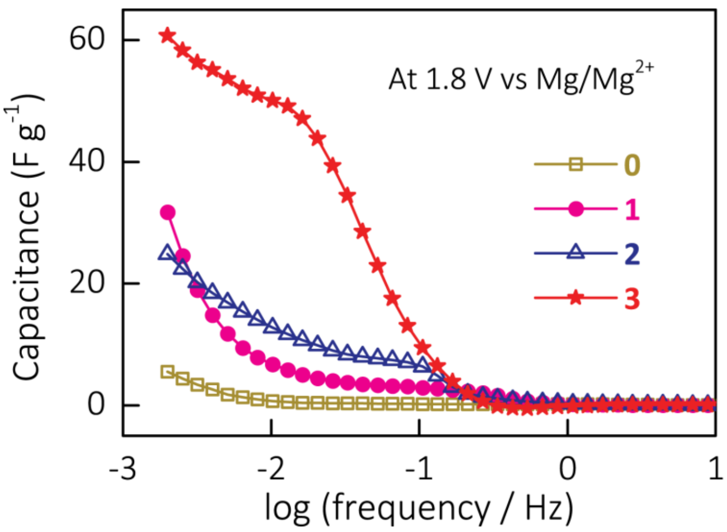

Supplementary Figure 9. Capacitance vs frequency plot from impedance measurements at a fixed potential of 1.8 V vs Mg/Mg^{2+} for stage **0–3** during the first discharge.

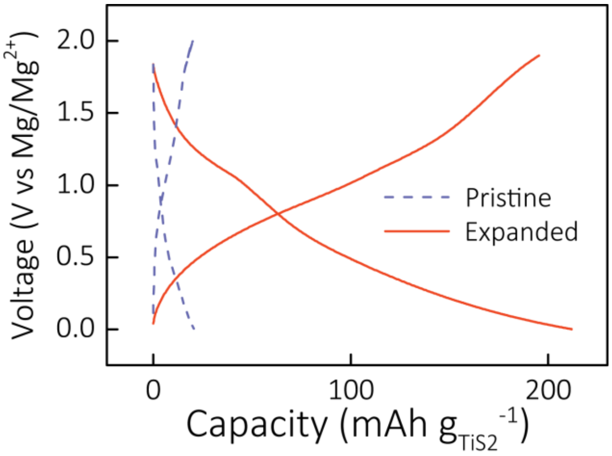

Supplementary Figure 10. Voltage profiles of pristine and expanded TiS_2 electrodes in APC electrolyte without $PY14^+$ ions.

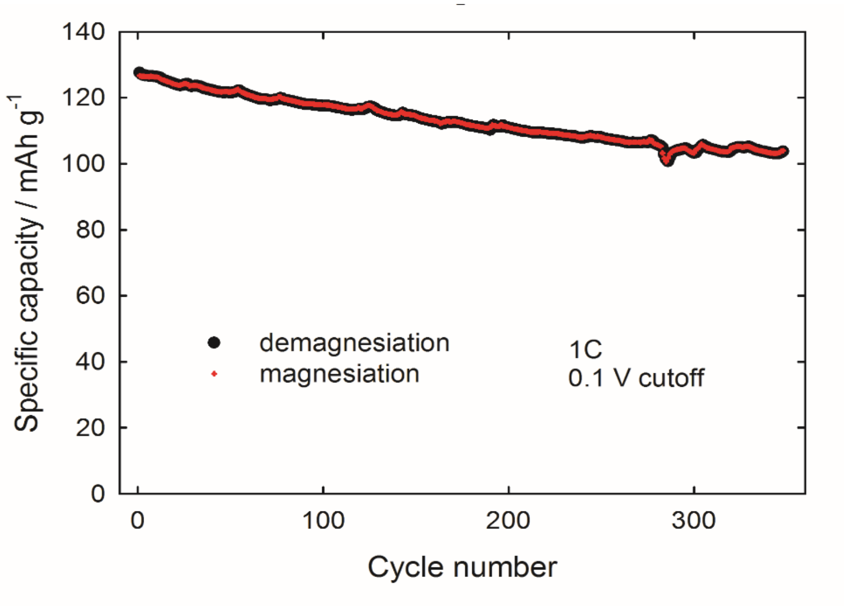

Supplementary Figure 11. Cycling stability of PY14$^+$-pillared, expanded TiS_2 in APC electrolyte without PY14$^+$ ions at 1C-rate (2.0–0.1 V). The cycling retention is 80% of the initial capacity after 350 cycles.

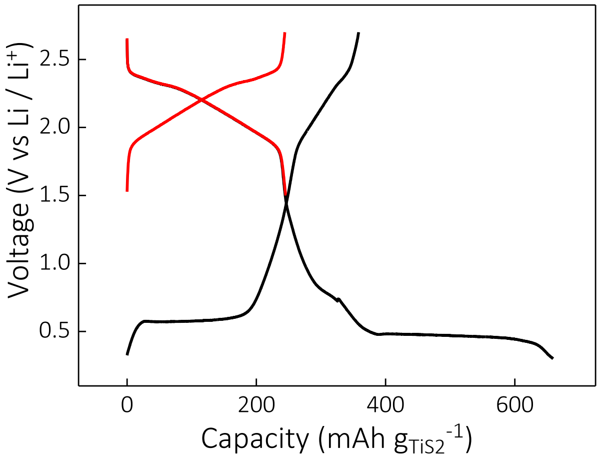

Supplementary Figure 12. Voltage profiles for Li intercalation in pristine TiS_2 with the discharge cutoff set to 1.5 (red) and 0.3 (black) V vs Li/Li^+. Li metal was used as anode with 1M $LiPF_6$ in EC:DEC as the electrolyte. The lowered cutoff voltage leads to more than two electron transfer per unit TiS_2 but with poor reversibility.

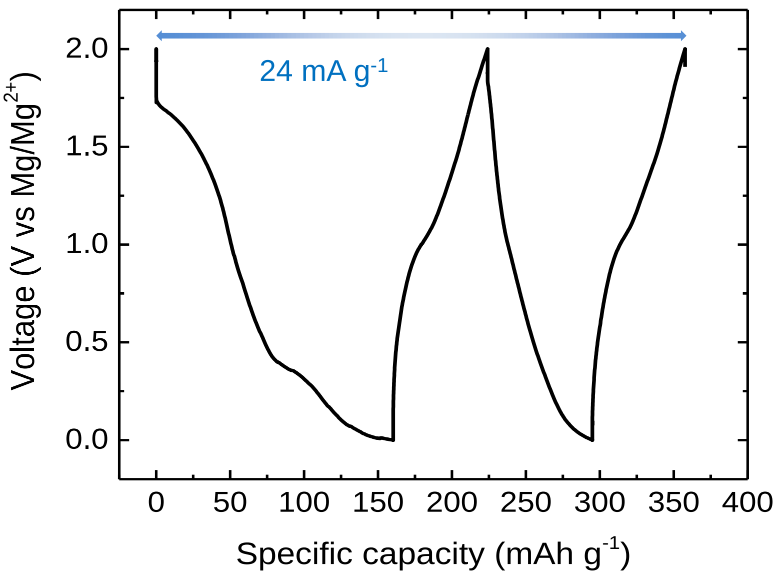

Supplementary Figure 13. The voltage profile of an expanded TiS_2 electrode stopped at stage **2** without further activation to stage **3**. Incomplete activation leads to low reversible capacity of 60 mAh g^{-1}.

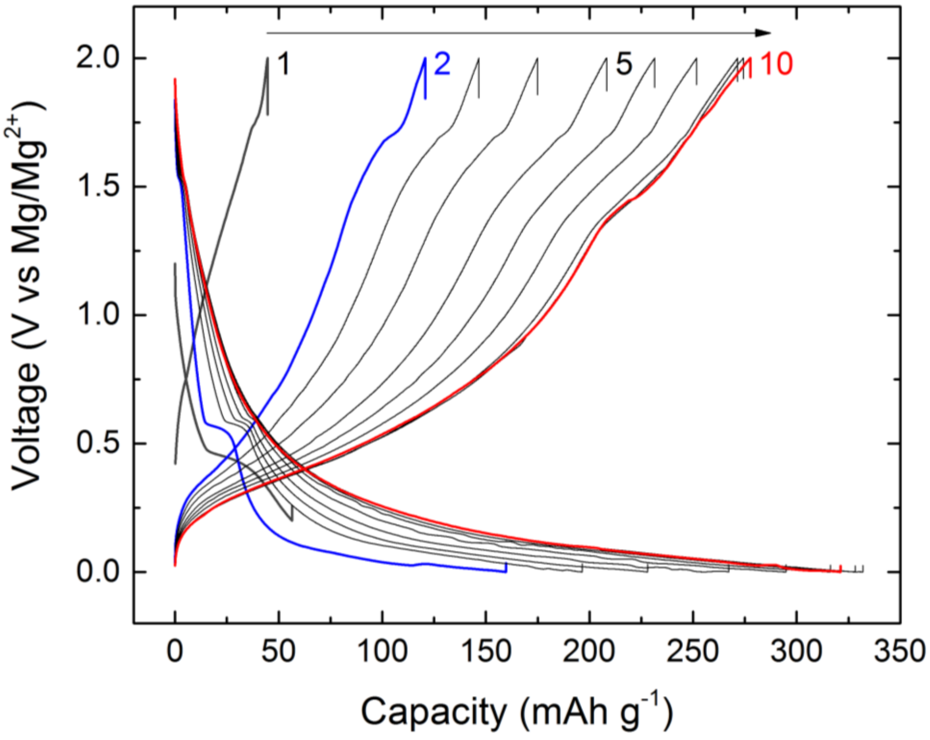

Supplementary Figure 14. Voltage profile of MoS_2 at 17 mA g^{-1} in the APC electrolyte that contains PY14Cl. The reversible capacity increases with cycling and reaches the maximum value of 277.7 mAh g^{-1} after 10 cycles.

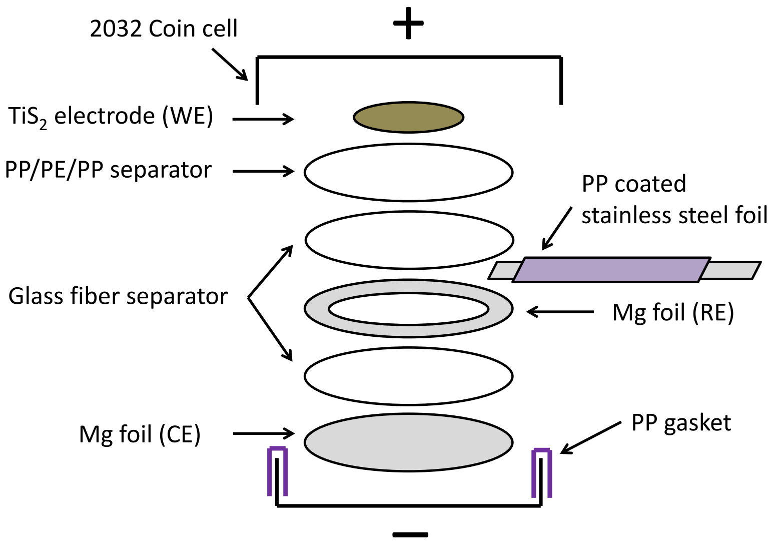

Supplementary Figure 15. Schematic of a three-electrode coin cell. Ring-shaped Mg metal foil was used as a reference electrode without blocking the working and counter electrodes. The reference electrode was connected out of the coin cell by polypropylene-coated stainless steel foil (50 μm and 350 μm thick before and after coating, respectively)[3]. Vacuum grease was applied on the joint for hermetic sealing of the cell.

Supplementary Tables

Supplementary Table 1. Electrochemical characteristics of the Mg-ion electrolyte (APC) with and without PY14Cl

	Voltage window (V)	Overpotential for Mg deposition (V)	Overpotential for Mg dissolution (V)	Coulombic efficiency (%)
APC	2.6	0.14	0.86	99.9
APC + 0.2 M PY14Cl	2.8	0.16	0.67	95.2

Supplementary Table 2. Interlayer spacing calculated from *in operando* XRD using Bragg's formula: $n\lambda = 2d_{00n}\sin\theta$

Stage	2θ (°)		Interlayer distance	n
	Measured	Corrected	d_{001} (Å)	
**0**	15.64	15.56	5.69	1
**1**	8.21	8.13	10.87	1
	16.39	16.31	10.86	2
**2**, **3**, **4**, **5**	4.82	4.74	18.63	1
	9.57	9.49	18.62	2
	14.34	14.26	18.63	3
	19.12	19.04	18.64	4

Supplementary Table 3. Characterization of expanded TiS_2 from stage **1** to **4** based on EDS, ICP-OES and NMR analysis

Stage	EDS	ICP-OES	NMR analysis
	Mg/Cl ratio	Mg/Ti ratio	PY14/THF ratio
**1**	N/A	0.02	4.18
**2**	0.8	0.46	3.15
**3**	1.1	1.0	1.30
**4**	0.8	0.14	1.36

Supplementary Table 4. TGA measurements and the compositions of stages **0** and **3** derived from TGA, ICP-OES, EDS, and NMR results

Stage	Weight (%) at 500 °C	Derived composition
**0**	82.9	TiS_2
**3**	55.1	$(MgCl)_{1.0}TiS_2[(PY14)_{0.20}(THF)_{0.16}]$

Supplementary Table 5. Gravimetric and volumetric capacity of pristine TiS_2, *ex*TiS_2, and Chevrel phase Mo_6S_8 at 25 °C

	Pristine TiS_2	*ex*TiS_2	Chevrel Mo_6S_8
Gravimetric capacity (mAh g^{-1})	20.5	173	100
Volumetric capacity (Ah L^{-1})	66	235	519

Supplementary References

1. Emly, A. & Van der Ven, A. Mg Intercalation in Layered and Spinel Host Crystal Structures for Mg Batteries. *Inorg. Chem.* **54**, 4394-4402 (2015).
2. Li, W., Reimers, J. N. & Dahn, J. R. *In situ* X-ray diffraction and electrochemical studies of $Li_{1-x}NiO_2$. *Solid State Ionics* **67**, 123-130 (1993).
3. Moshurchak, L., Dahn, J., Obrovac, M. & Christensen, L. Design and Use of Three Electrode Coin Cells for Studying Redox Shuttles. *ECS Meeting Abstracts* **MA2005-02**, 218 (2006).
